# Supplementary material for: Iberian Margin surface ocean cooling led freshening during Marine Isotope Stage 6 abrupt cooling events
Source: Nat Commun. 2023 Sep 4;14:5390. doi: 10.1038/s41467-023-41142-8 (PMC10477208; doi:10.1038/s41467-023-41142-8)
Supplement: Supplementary file 1 — Supplementary information [file 41467_2023_41142_MOESM1_ESM.pdf]

Supplementary for “Iberian Margin surface ocean cooling led freshening during Marine Isotope Stage 6 abrupt cooling events”

Hongrui Zhang[1]*, Yongsong Huang[2], Reto Wijker[1], Isabel Cacho[3], Judit Torner[3], Madeleine Santos[1], Oliver Kost[1], Bingbing Wei[4], Heather Stoll[1]

1. Geological Institute, ETH Zürich, 8092 Zürich, Switzerland;
2. Department of Earth, Environmental and Planetary Sciences, Brown University, Providence, RI 02912, USA;
3. Grup de Recerca Consolidat en Geociències Marines, Department de Dinàmica de la Terra i de l’Oceà, Universitat de Barcelona, Barcelona, Spain;
4. Alfred Wegener Institute, Helmholtz Center for Polar and Marine Research, Bremerhaven, 27570, Germany
* Correspondence and requests for materials should be addressed to HZ

Email: zhh@ethz.ch

This document includes:

Figure S1. Gas chromatographic results in two samples with low and high $C_{37:4}$%.

Figure S2. Sea surface temperature during MIS 6 in different site on the Iberian Margin

Figure S3. Reworked fossils and oxygen isotope of deep ocean.

Figure S4. The oxygen isotope and Mg/Ca ratio measurements.

Figure S5. Comparison of alkenone-based SST and Mg/Ca SST on the Iberian Margin

Figure S6. Cross plot among $C_{37:4}$%, $\delta^{18}O_{sw}$ and alkenone-based SST.

Table S1. The endmember of mixing calculations in Figure 2.

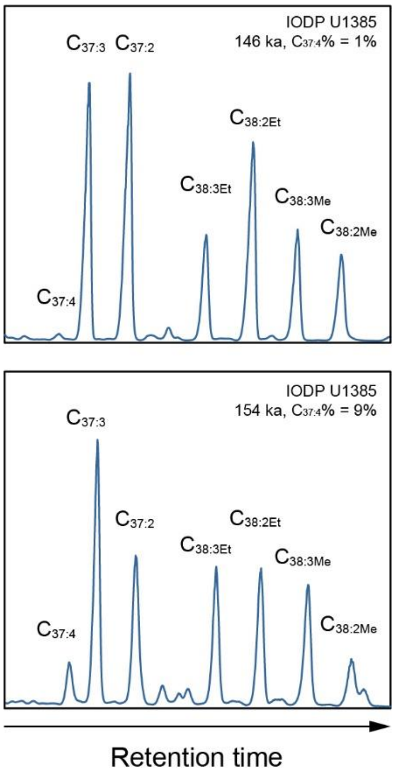

Figure S1. Gas chromatographic results in two samples with low and high $C_{37:4}$%.

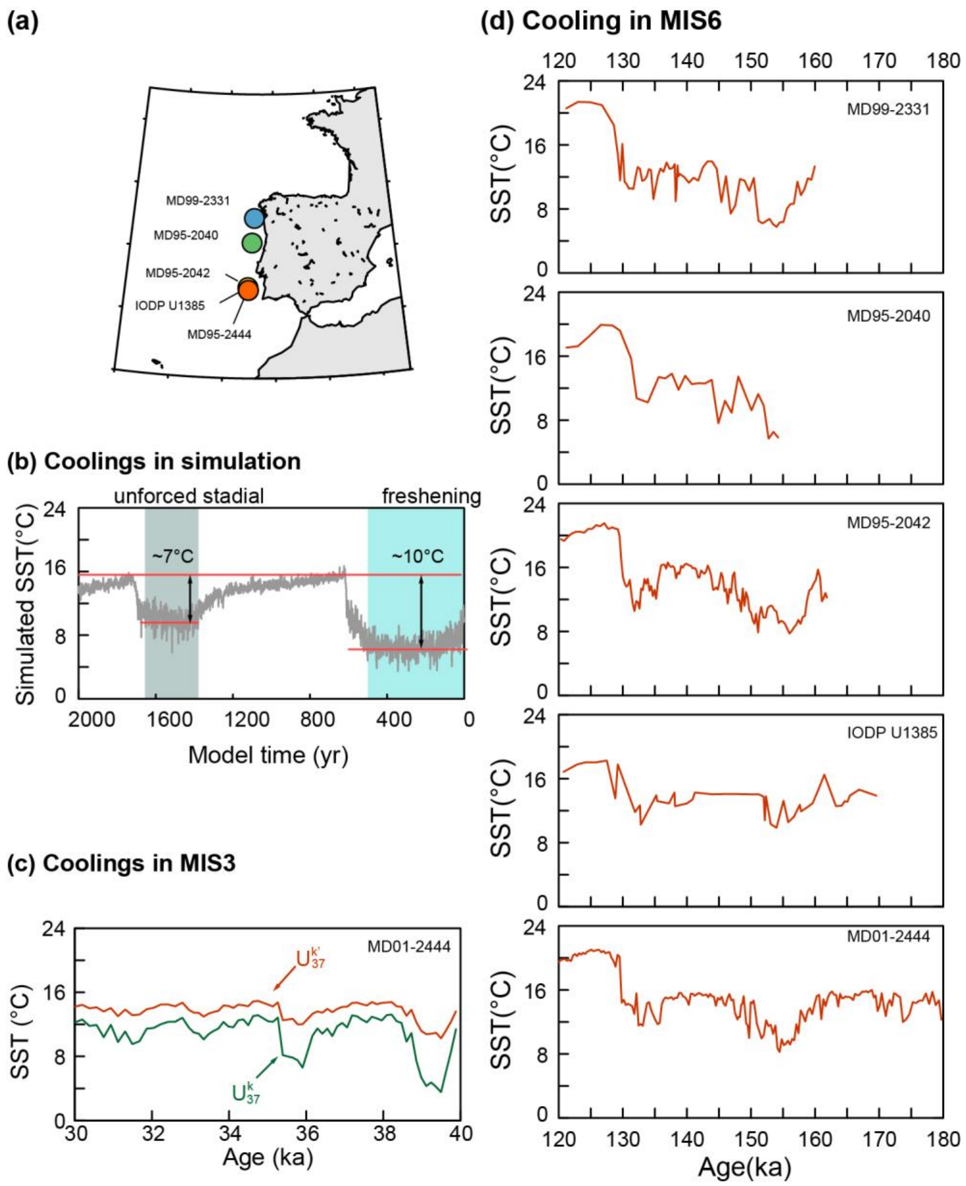

Figure S2. SST by $U^{k'}_{37}$ along the Iberian Margin. (a) Position of each site. (b) Simulated cooling events on the Iberian Margin (37.97°N, 10.2°W) with forced freshwater (light blue) and without freshwater (grey)[1]. (c) SST reconstructed by alkenone thermometers in the MIS 3. (d) The data in site MD99-2331, MD95-2040 and MD95-2042 are from [2]. The results in site IODP U1385 are from this study and [3]. Red lines are SST reconstructed by $U^{k'}_{37}$ with Bayesian calibration. Green lines are SST reconstructed by U^{k}_{37}[4].

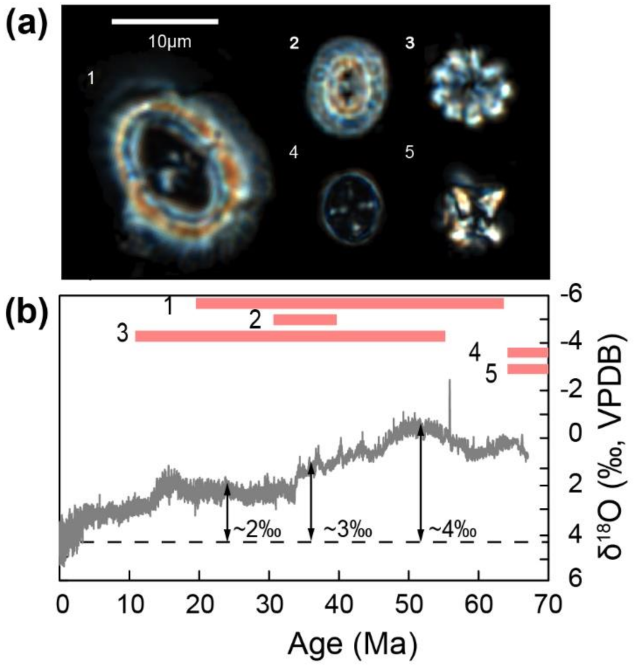

Figure S3. Reworked fossils and oxygen isotope of deep ocean. (a) fossils in circular polarized microscope. From 1 to 5: *Chiasmolithus* spp., *Reticulofenestra filewiczii, Discaster deflandrei*, *Chiastozygus stylesii* and *Micula staurophora*. **(b)** The stack of deep ocean (benthic foraminifera) oxygen isotope[5]. The pink bars represent the occurrence time of fossils (Nannotax3).

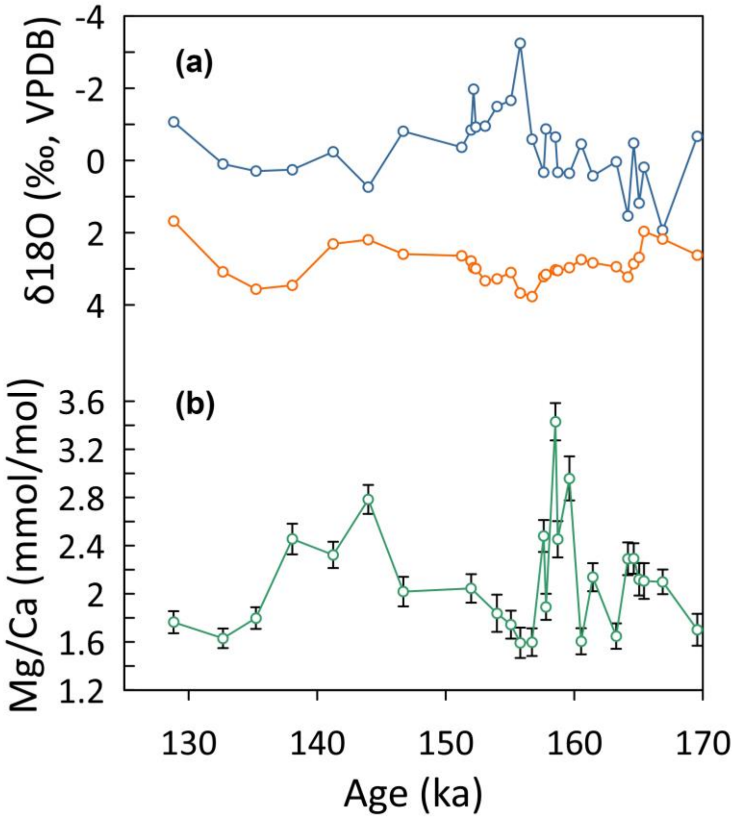

Figure S4. The oxygen isotope and Mg/Ca ratio measurements. (a) Oxygen isotope of planktonic foraminifera, *Globigerina bulloides* (orange dots) and fine fraction (<63µm, blue dots). **(b)** The Mg/Ca ratio of *G. bulloides*. The error bars represent the standard deviation of measurement.

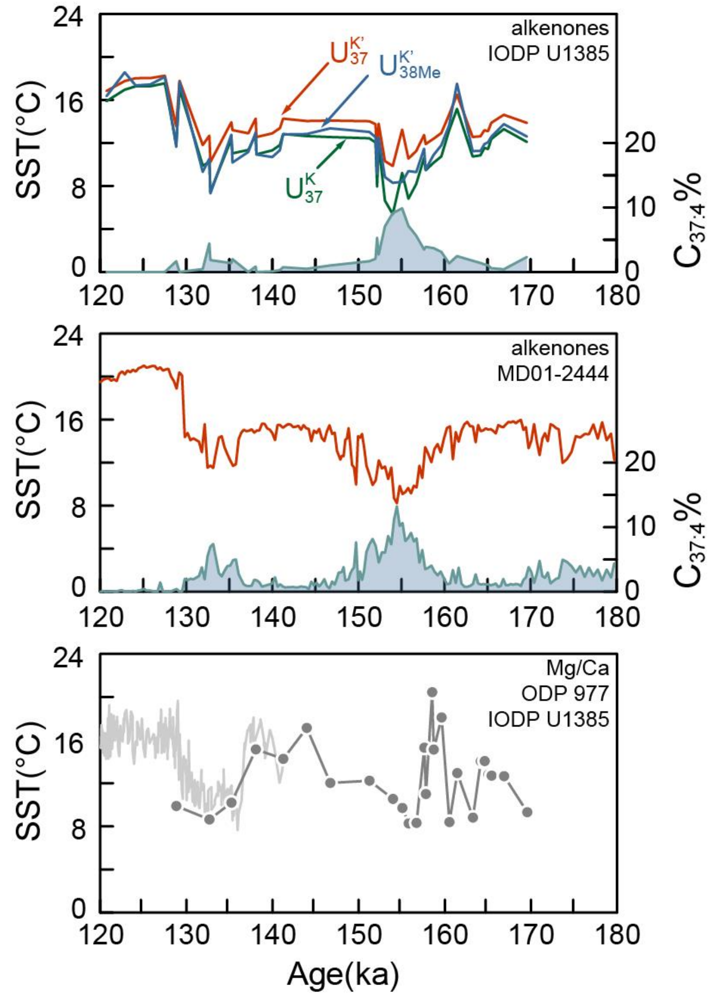

Figure S5. Comparison of alkenone-based SST and Mg/Ca SST on the Iberian Margin. Red lines are SST reconstructed by $U_{37}^{K\prime}$ with Bayesian calibration [6]. Green lines are SST reconstructed by U_{37}^{K} by Rosell-Melé [4]. Blue lines are SST reconstructed by $U_{38Me}^{K\prime}$ by Novak, et al. [7]. The shaded areas are $C_{37:4}$%. Grey lines are Mg/Ca of *G. bulloides* in site ODP 977 [8] and grey dots are new records in this study.

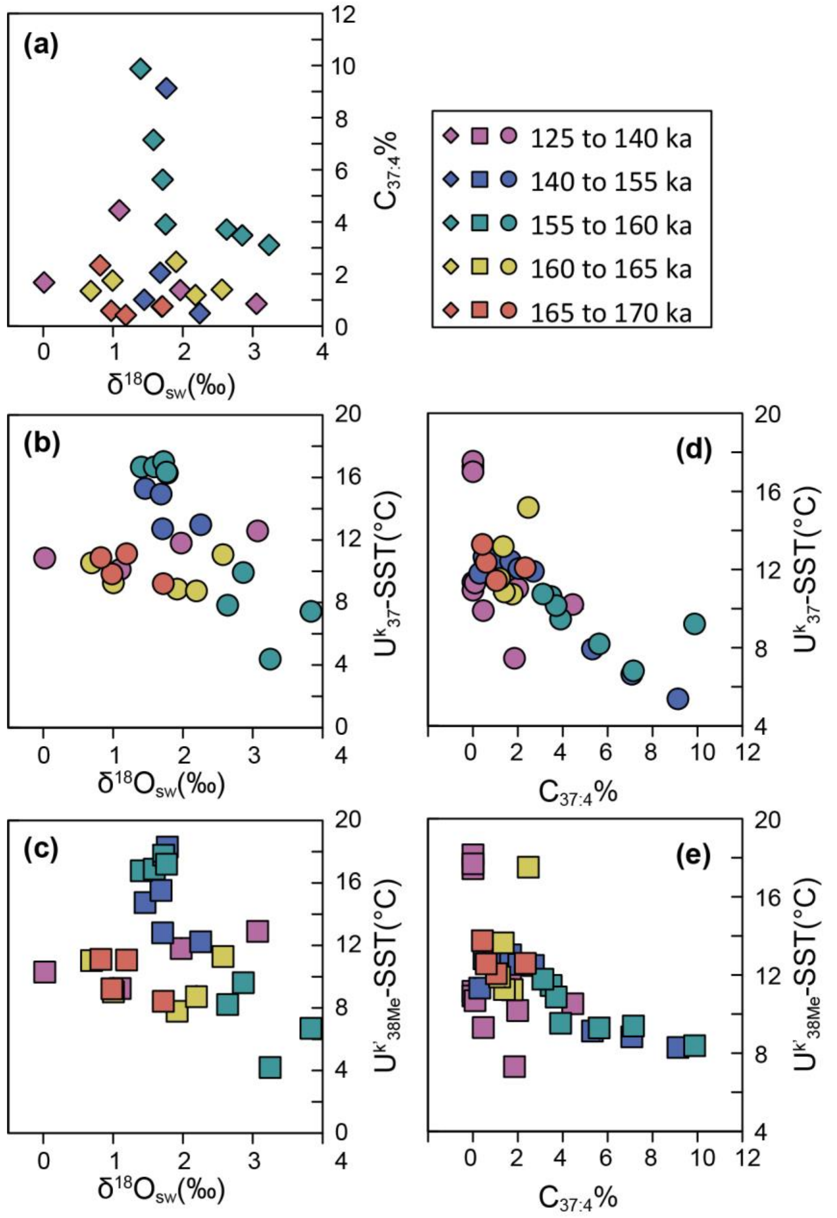

Figure S6. Cross plot among $C_{37:4}$%, $\delta^{18}O_{sw}$ and alkenone-based SST. **(a)** Cross plot between $C_{37:4}$% and $\delta^{18}O_{sw}$. **(b-e)** Cross plot among $C_{37:4}$%, $\delta^{18}O_{sw}$ and SST estimated by U_{37}^{K} and $U_{38Me}^{K\prime}$. The squares and dots in b-e represent SST reconstructed by $U_{38Me}^{K\prime}$ and U_{37}^{K}, respectively.

Table S1. The endmember of mixing calculations in Figure 2.

	Group I		Group II		Group III warm		Group III cold	
	$C_{37:4}$%	$C_{38ME:}$:C_{38ET}	$C_{37:4}$%	$C_{38ME:}$:C_{38ET}	$C_{37:4}$%	$C_{38ME:}$:C_{38ET}	$C_{37:4}$%	$C_{38ME:}$:C_{38ET}
**Figure 2c**	40	0.2	--	--	0	0.3-2	--	--
**Figure 2d**	--	--	15-40	0	0	0.3-1	--	--
**Figure 2e**	--	--	--	--	0	0.3-1	10-40	2

Supplementary references

1 Pedro, J. B. *et al.* Dansgaard-Oeschger and Heinrich event temperature anomalies in the North Atlantic set by sea ice, frontal position and thermocline structure. *Quaternary Science Reviews* **289**, doi:10.1016/j.quascirev.2022.107599 (2022).

2 Davtian, N., Bard, E., Darfeuil, S., Ménot, G. & Rostek, F. The Novel Hydroxylated Tetraether Index RI-OH′ as a Sea Surface Temperature Proxy for the 160-45 ka BP Period Off the Iberian Margin. *Paleoceanography and Paleoclimatology* **36**, doi:10.1029/2020pa004077 (2021).

3 Martrat, B. *et al.* Four climate cycles of recurring deep and surface water destabilizations on the Iberian margin. *Science* **317**, 502-507 (2007).

4 Rosell-Melé, A. Interhemispheric appraisal of the value of alkenone indices as temperature and salinity proxies in high-latitude locations. *Paleoceanography* **13**, 694-703, doi:10.1029/98PA02355 (1998).

5 Westerhold, T. *et al.* An astronomically dated record of Earth's climate and its predictability over the last 66 million years. *Science* **369**, 1383-1387, doi:10.1126/science.aba6853 (2020).

6 Tierney, J. E. & Tingley, M. P. BAYSPLINE: A New Calibration for the Alkenone Paleothermometer. *Paleoceanography and Paleoclimatology* **33**, 281-301, doi:10.1002/2017pa003201 (2018).

7 Novak, J. *et al.* UK′38ME Expands the linear dynamic range of the alkenone sea surface temperature proxy. *Geochimica et Cosmochimica Acta* **328**, 207-220, doi:10.1016/j.gca.2022.04.021 (2022).

8 Torner, J. *et al.* Ocean-atmosphere interconnections from the last interglacial to the early glacial: An integration of marine and cave records in the Iberian region. *Quaternary Science Reviews* **226**, 106037, doi:10.1016/j.quascirev.2019.106037 (2019).
